# Work aggravated asthma in Great Britain: a cross-sectional postal survey

**DOI:** 10.1017/S1463423618000063

**Published:** 2018-04-12

**Authors:** Lisa Bradshaw, Jade Sumner, Julian Delic, Paul Henneberger, David Fishwick

**Affiliations:** 1 Centre for Workplace Health, Health and Safety Executive, Buxton, Derbyshire, UK; 2 Health and Safety Executive, Redgrave Court, Bootle, Merseyside, UK; 3 Respiratory Health Division, National Institute for Occupational Safety and Health (NIOSH), Centers for Disease Control and Prevention (CDC), Morgantown, WV, USA

**Keywords:** asthma, occupational health, work aggravated, work related

## Abstract

**Objective:**

Work aggravated asthma (WAA), asthma made worse by but not caused by workplace exposures, can have a negative impact on personal, social, financial and societal costs. There is limited data on prevalence levels of WAA in Great Britain (GB). The objective of this study was to estimate the prevalence of WAA in GB, and to assess its potential causes.

**Materials and methods:**

A cross-sectional postal questionnaire study was carried out. A total of 1620 questionnaires were sent to three populations of adults with asthma. The questionnaire recorded; demographic details, current job, self-reported health status, presence of asthma and respiratory symptoms, duration and severity of symptoms and medication requirements. Questions relating to work environment and employers’ actions were included, and each participant completed an assessment of health-related quality of life using the EuroQol Research Foundation EQ-5D.

**Results:**

There were 207 completed questionnaires; response rates were 6% primary care, 45% secondary care and 71% Asthma UK. This represented a 13% overall response rate. Self-reported prevalence of WAA was 33% (95% CI 24.4–41.6%). In all, 19% of workers had changed their job because of breathing problems. Workers with WAA reported higher levels of work-related stress. Quality of life using the EQ-5D utility index was lower in those with WAA.

**Conclusion:**

WAA is a common problem in asthmatics in GB. This result is in keeping with international prevalence rates. Further research could assist the understanding of the most significant aggravants to asthma at work and help define appropriate interventions by workplaces.

## Background

Workers with asthma often complain that their symptoms worsen at work. These symptoms are likely to represent work-related asthma, which comprises two diagnoses; either occupational asthma (OA) itself or WAA. Both can cause significant disruption to healthy working lives, and have personal, prescription, quality of life, healthcare, financial and societal costs (Cannon *et al*., 1995; Lowery *et al*., 2007; Mazurek *et al*., 2011; Knoeller *et al*., 2012; 2013). These can all be minimised by making the correct diagnosis, and subsequent proactive management.[Boxed-text boxed-text1]

What this paper adds

∙ International prevalence of work aggravated asthma (WAA) is estimated at 21% of all asthma (range 13–58%). Currently, there is no published data on the prevalence in Great Britain (GB). ∙ This study investigates WAA prevalence for the first time in three groups of individuals in GB. ∙ Asthma symptoms that worsen at work (WAA) are commonly reported by workers in GB. The estimate of 33% of those with asthma complaining of work-related symptoms is in line with international prevalence. ∙ Future research and clinical work could identify and improve the understanding of the most significant aggravants to asthma at work. This would undoubtedly help define appropriate interventions for workers with asthma.

Whilst OA is relatively well understood (Fishwick, 2008; British Occupational Health Research Foundation, 2010; Fishwick, 2016), where sensitisation to an inhaled agent such as flour is assumed, little is known about WAA. WAA describes the situation where asthma is aggravated, but not caused, by workplace exposures (Fishwick *et al*., 2013), and is now identified to be relatively common. Many different types of aggravants have been described (Fishwick, 2014) including general inhaled exposures (such as dust, smoke, vapours, fumes, gases, mists and sprays), inhaled allergens (such as flour dust), physical environmental factors (such as extremes of workplace temperature or humidity), physical activity at work and additional considerations such as workplace stress.

Symptoms of WAA may begin several minutes or hours after relevant exposures occur at work, and can be either short lived or consistent over a period of time (Tarlo *et al*., 2009). Workers may also describe reduced levels of medication needed to control asthma when not at work, increased use of reliever medication at work, and/or improvement in symptoms when harmful workplace exposures are reduced or eliminated.

A previous literature review by the American Thoracic Society has estimated (Henneberger *et al*., 2011) that a median of 21.5% of adults with asthma complained of symptoms that were worse at work. This figure was derived from 12 studies, with a range of estimates between 13% and 58%; although no GB prevalence estimates were identified for inclusion. Three of the 12 studies used more objective criteria that went beyond self-reports alone, and the median prevalence among those three was 14%. The authors suggested that the definition of WAA should include the presence of pre-existing or concurrent asthma, a temporal relationship between asthma and work, conditions at work that could aggravate asthma and finally that occupational asthma due to sensitisation was unlikely.

## Objective

We therefore present the findings of the first GB-based study where the primary aim was to estimate the prevalence and extent of WAA, and to assess its potential causes.

## Materials and methods

A cross-sectional questionnaire study was carried out in three populations of adults with asthma. These were a (i) primary care population, (ii) a secondary care, hospital recruited, population of patients with asthma and (iii) a population recruited from the major GB Third Sector organisation working as advocates for patients with asthma.

The primary care population was identified by working with the relevant Clinical Commissioning Groups; who identified two general practices in South Yorkshire, GB, willing to participate. All 1400 patients included in their asthma registers, between the ages of 18–65 years old, were invited to participate. The secondary care hospital-based population was identified using a pre-existing National Health Service (NHS) research database held by an NHS Trust in South Yorkshire. All 151 asthmatic patients registered on this database were invited to participate, this database consisted of name, hospital number, address and diagnosis. We were unable to filter by date of birth so it included all over 18-year olds with a diagnosis of asthma.

Questionnaires were posted to participants in February 2015 and non-responders were sent a reminder letter and a further hard copy of the questionnaire four weeks after the original mail out. This asked the individual to reconsider completing the study questionnaire. If no questionnaire was returned, or telephone message received, after three months from the initial mail out, non-participation was assumed.

The Third Sector organisation invited the members of its pre-registered Research and Policy Group, comprising volunteer patients GB with asthma, who were currently employed, to participate. In addition, its Facebook and Twitter feeds were used to highlight the study. All potentially interested individuals were asked to contact the study team for a study questionnaire during January and February 2015. These volunteers were geographically spread across GB.

The questionnaire itself was developed to record demographic details, current job where relevant, self-reported health status, the presence of asthma and respiratory symptoms, their duration, severity and medication requirements. Questions relating to the work environment and any employers’ actions were also included, and each participant completed an assessment of health-related quality of life using the EuroQol Research Foundation EQ-5D. The EQ-5D consists of two parts; a descriptive section and a visual analogue scale. The descriptive section comprises five dimensions: mobility, self-care, usual activities, pain/discomfort and anxiety/depression. Each dimension has three levels: no problems, some problems, extreme problems. The second section records the respondent’s self-rated health on a vertical, visual analogue scale. This assessment tool has been validated in a diverse patient population in six countries, including eight patient groups with chronic conditions (cardiovascular disease, respiratory disease, depression, diabetes, liver disease, personality disorders, arthritis, stroke) and a student cohort.

In order to identify those who were at increased risk of occupational asthma *per se*, as this study was not able to reliably identify this diagnosis using the questionnaire alone, a question was included relating to relevant potential asthmagen exposures at work when their breathing problems began. The response to this question was used to identify an *a priori* group that may have been exposed to an agent able to cause their asthma and could thus be at an increased risk of OA. Data from the Health and Safety Executive (HSE) sponsored surveillance of work-related and occupational respiratory disease scheme (2009–2014) were used to define the high risk occupations; bakers/flour, food processors, cleaners, vehicle sprayers, and assemblers and electronics workers.

A question was also included from the Work Productivity and Activity Impairment Questionnaire (WPAIQ); which is a validated six-question survey used to measure health-related work productivity loss for the employed population due to health problems. The response from the WPAIQ question was used to benchmark how much the presence of asthma had interfered with work productivity, effectively a measure of presenteeism, over the previous seven days. In addition, the British Thoracic Society (BTS) stepwise approach to asthma classification was used to benchmark asthma severity, and the Computer Assisted Structured Coding Tool (CASCOT) program, allowing classification of occupations to standards developed by the UK Office for National Statistics, was used to code current job in to one of the nine major categories.

The questionnaire underwent a pilot application, where five existing patients with asthma were asked to feedback their views. Quantitative data were entered and checked using the SPSS Statistical Package (SPSS 14.0; SPSS Inc., Chicago, IL, USA). All categorical and continuous variables were subject to descriptive analysis, followed by a combination of univariate analyses to assess differences of proportions and means between those with and without work aggravated asthma. Continuous variables were assessed for normality before tests of significance; taken at the 5% level.

Ethics agreement was gained from the NHS Research Ethics Committee (reference numbers 14/NE/1061 and 14/SC/1296).

## Results

A pilot exercise with the questionnaire did not lead to any changes in the content. A total of 1630 postal questionnaires were sent to potential participants. The breakdown of this between study populations was as follows; primary care population *n*=1400, secondary care population *n*=151 and Asthma UK population *n*=79.

A total of 207 questionnaires were received. Response rates were as follows; primary care population 83/1400 (6%), secondary care population 68/151 (45%) and Asthma UK population 56/79 (71%). This represented a 12.7% overall response rate. In all, 13 individuals were either out of the country, or the questionnaire was returned to sender; revising the overall response rate to 12.8%; 142 of the 207 (69%) currently worked. The mean age was 47.7 years (SD 14.7), 136 were female (66%), 19 (9%) were current smokers, 114 (55%) had never smoked, and 199 (96%) had a self-reported diagnosis of asthma. The following analysis is restricted to those 136 respondents who currently worked and also self-reported a diagnosis of asthma. Those individuals who did not self-report a diagnosis of asthma were excluded from the final analysis, these individuals may have been in advertently left on the asthma register after a diagnosis of asthma had been refuted.

In terms of asthma severity, patients who supplied asthma medication details (*n*=100) were benchmarked according to their BTS asthma step (British Thoracic Society, Scottish Intercollegiate Guideline Network, 2003); 9% were at step 1 (the least severe), 26% at step 2, 41% at step 3, 19% at step 4 and 5% at step 5 (the most severe); 4% of individuals were not currently taking any asthma medication. Self-reported health status in this group was generally good; with 94 (69%) rating good, very good or excellent general health.

Current self-reported jobs of the respondents (three with data missing excluded) were categorised by CASCOT as follows; managers, directors & senior officials 14 (10%), professional occupations 9 (7%), associate professional & technical occupations 33 (24%), administration & secretarial occupations 21 (15%), skilled trades occupations 8 (6%), caring, leisure & other service occupations 13 (10%), sales & customer service occupations 9 (7%), process plant & machine operatives 4 (3%) and elementary occupations 22 (16%). In all, 14 workers (10%) were in an *a priori* defined high-risk job for occupational asthma.

Asthma symptoms were self-reported to be worse at work in 33.3% (95% CI 24.4–41.6%) of those with asthma who worked, based on 126 responses. This estimate remained identical when only those in low-risk jobs for occupational asthma were considered. The majority (78%) of workers who self-reported symptoms consistent with WAA felt that they used more asthma medication on work days in comparison with rest days. The full relationship between reported asthma symptoms and medication use at work is shown in Table 1, and it is clear that those with self-reported WAA symptoms appeared to increase their reliever medication at work as a consequence. Likewise, it appeared that those patients without self-reported worsening of their asthma symptoms at work did not, in general, require increasing doses of treatment for their work days.

**Table 1:** Asthma symptoms and medication use at work

	Is asthma medication used the same, less or more on work days compared with days away from work?		
	Same [*n* (%)]	Less [*n* (%)]	More [*n* (%)]
Are your asthma symptoms the same, better or worse at work than days away from work?^a^			
Same	61 (80%)	2 (3%)	13 (17%)
Better	0 (0%)	4 (80%)	1 (20%)
Worse	7 (17%)	2 (5%)	32 (78%)

a Based on 125 responses, 11 with missing data

**Figure 1:**
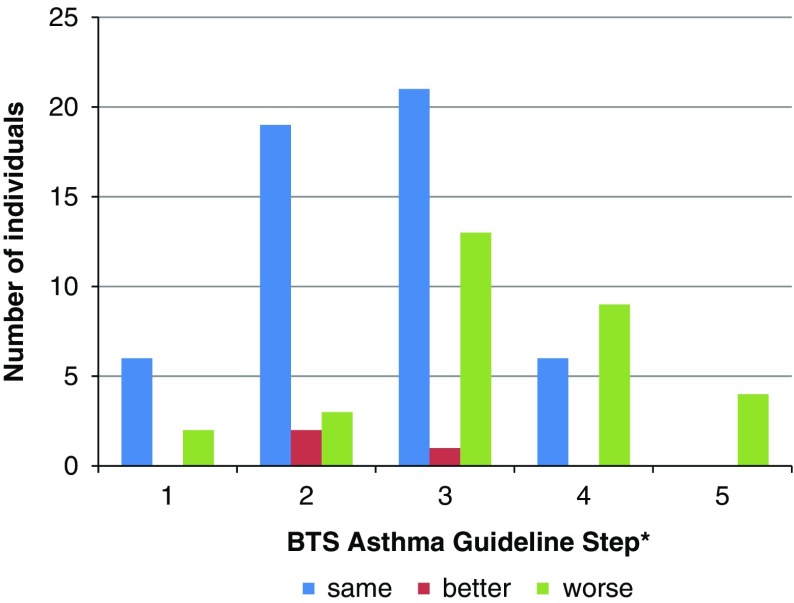
The relationship between British Thoracic Society (BTS) asthma severity step and the presence of self-reported symptoms consistent with work aggravated asthma *The BTS asthma steps range from 1 for least severe to 5 for most severe asthma.

Figure 1 illustrates the relationship between asthma severity and self-reported WAA symptoms, and it is seen that those with more severe asthma tended to have higher levels of symptoms consistent with WAA. Improvement in asthma symptoms at work was confined to respondents with asthma in BTS steps 2 and 3.

Table 2 illustrates the levels of potential causes for work-related asthma symptoms. It is evident that the presence of work-related symptoms was associated with increased self-reported dusts, fumes and gases exposure at work, strenuous activity at work and stress at work. Only the latter was significantly over reported in those with work-related symptoms.

**Table 2:** Potential causes of asthma symptoms worsening at work broken down by presence or absence of work-related asthma symptoms

	Asthma symptoms at work		
Self-reported presence of potential causes of asthma symptoms at work	Same *n* (%)	Better *n* (%)	Worse *n* (%)
Dust exposure?^a^			
Yes	38 (58%)	1 (2%)	26 (40%)
No (*n*=123)	38 (66%)	4 (7%)	16 (27%)
Strenuous activity at work?^b^			
Yes	19 (58%)	1 (3%)	13 (39%)
No (*n*=100)	43 (64%)	3 (5%)	21 (31%)
Stress at work?^c^			
Yes	43 (56%)	3 (4%)	31 (40%)
No (*n*=100)	19 (83%)	1 (4%)	3 (13%)
Extreme temperatures at work?^d^			
Yes	21 (51%)	2 (5%)	18 (44%)
No (*n*=122)	56 (69%)	3 (4%)	22 (27%)

a Are you exposed to vapours, gases dust or fumes at work? *P*=NS.

b Do you carry out any strenuous physical activity at work as part of your job? *P*=NS.

c Do you ever feel stressed at work? *P*=0.052. Chi squared comparing worse with (better and same) combined, *P*=0.16.

d Are you exposed to extreme temperatures at work? *P*=0.149. Chi squared comparing worse with (better and same) combined, *P*=0.063.

Various consequences of the presence of work-related symptoms were assessed, and included sickness absence, self-reported well-being and productivity at work, and the previous requirement for job change because of breathing problems. Sickness absence levels relating to breathing problems were high; based on those with asthma and working (*n*=132), a mean of 1.9 (range 0–28) days was lost over the preceding four weeks and 12.3 (range 0–365) days over the preceding 12-month period.

**Figure 2:**
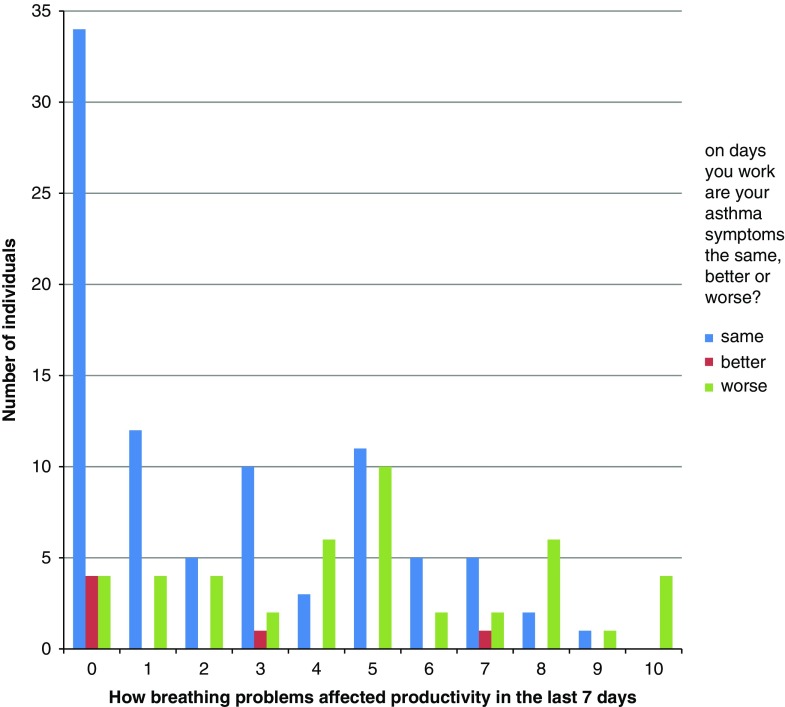
Relationship between work-related asthma symptoms and self-reported productivity at work influenced by the presence of asthma (The scale on the *x*-axis ranges from 0 to 10 with 0 being asthma has no effect on productivity and 10 being asthma has a severe effect on productivity.)

Similarly, responses to the question concerning work productivity suggested that the presence of asthma impaired the ability, in certain cases, to carry out normally productive work, suggesting that asthma, and in particularly work-related asthma, caused significant presenteeism (presenteeism can be defined as workers who come to work but do not perform to their best ability because of illness making them less productive). These data are shown in Figure 2.

In addition, approximately one fifth (*n*=24, 19.5%) of those with self-reported asthma noted that a job change had been previously necessary because work had affected their breathing. Finally, the majority of workers reported that their employer did not put control measures in place to control exposures that may affect their breathing. Notably, of the 34 workers who reported work-related symptoms and also responded to the question relating to employer intervention, 23 (68%) reported that the employer did not do anything to help control exposures at work that may be affecting their breathing.

The summary EQ-5D utility index, used as an overall assessment of health-related quality of life was lower in those with work-related symptoms (mean 0.74, SD 0.22 ) in comparison with those without (mean 0.79, SD 0.20). This difference did not reach statistical significance (Mann–Whitney *U* test, *P*=0.159).

## Discussion

### Main findings

Symptoms of asthma that worsen at work are commonly reported by differing GB populations of workers with asthma. The estimate of 33% of those with asthma complaining of these work-related symptoms is in the previous range of prevalence rates identified by Henneberger *et al*. These work-related asthma symptoms additionally appeared to be associated with both an increased severity of asthma as judged by their BTS step, although work-related symptoms were also present in some patients at less severe BTS steps 1 and 2, and increased reliever medication taken at work; not seen in those without these symptoms. Exclusion of those in high-risk jobs for occupational asthma, where exposure to sensitisers was *a priori* decided to be more likely did not significantly alter this estimate.

In addition, those who reported work-related symptoms had greater levels of self-reported work-related stress. In some cases, this could be due to Effort-Reward Imbalance (ERI). The principle of the ERI Model (Siegrist *et al*., 1986) is that an imbalance between high efforts and low rewards leads to sustained strain reactions. It suggests that high efforts in combination with low rewards increase the risk of poor health and also that a high level of overcommitment to work may increase the risk of poor health. A review of 45 empirical studies reported that workers reporting both ERI and a high level of overcommitment have an even higher risk of poor health (van Vegchel *et al*., 2005).

The findings of this study, we believe, offer useful new insights into the nature of work-related asthma symptoms in the GB that compliment and add to the existing literature. It was evident that, in this group, reported inhaled exposures, and exercise levels, did not appear to separate those with and without work-related symptoms. This may be a more generalisable finding, or may relate to the relatively limited nature of the study population. Self-reported stress at work was, however, associated with work-related symptoms. The cross-sectional nature of this study did not allow further understanding of this finding. Namely, was self-reported stress a consequence of worsening asthma, or worsening asthma a consequence of self-reported stress? This appears to be an important area to study further, in so far as a better understanding of these links will help develop useful interventions for both primary care and the workplace.

### Strengths and limitations of this study

This study has a significant set of weaknesses. First, the overall response rate was very low, participation rates for epidemiologic studies have been declining over the past 30 years (Bradburn, 1992; Groves, 2004), and even more so in recent years (Curtin *et al*., 2005; Nohr *et al*., 2006) this not only applies to academic studies but profit making organisations (Tortora, 2004) and government departments have also reported this (Forthofer, 1983). Although, it is recognised that such populations are challenging to reach with questionnaire-based studies, and an additional reminder was sent in order to improve this where possible. Whilst the differing nature of each population was felt important to create a varied final study population, the primary care population in particular appeared to be difficult to reach using this method. The letter of invitation did not include the letterhead of the General Practitioner, it came directly from the researchers and this may have had an impact on response. The subjects had no previous interaction with the researchers and this may have influenced the low participation rates within the primary care group. Future work should consider how best to optimise response in this group of subjects, and in particular whether more central involvement of their primary care physician might assist in higher participation rates. The secondary care population were taken from an adult database of individuals with asthma. This database could not be filtered for age, it is therefore possible that some of the none responders were retired or were no longer in employment so may have felt that the study was not relevant to them. Nevertheless, the BTS asthma severity profile of the participants broadly matched the GB age-related profile identified by Neville *et al*. (2001), with perhaps a slight predominance of step 3 cases in our population when using the 31–64 years old age groups for comparison.

Second, we could not be confident that we had excluded those with a diagnosis of occupational asthma itself, with ongoing exposure to a causative agent causing work-related symptoms. We attempted to exclude those at higher risk of this alternate diagnosis by developing a high-risk occupational group. When this high-risk group were excluded, broadly similar findings resulted.

Finally, with the low response rate we cannot be sure of the generalisability of the results. In addition, ethnic diversity was limited therefore caution is warranted when generalising these findings to the wider population. In terms of limitations, the sample may not be representative of the whole working population with asthma, even though we did attempt to recruit participants from three different databases to gain a more representative cohort. We also have to be aware that this study may have had participation bias with more people responding who had work-related asthma symptoms; however, our prevalence findings are in line with other international studies.

The main strength of the study was the use of a structured questionnaire reducing the potential for interviewer bias. This approach will also allow the study to be replicated in different areas or over time with the production of comparable findings. The use of a validated quality of life questionnaire also allows the data collected to be compared with other cohorts of workers.

### Quality of life in WAA

If the suggested, but not significantly, reduced health-related quality of life is a true finding, assuming this study to have been underpowered to identify this true difference, this it could reflect either the increased asthma severity seen in those with work-related symptoms, or perhaps be a specific effect of limitations posed on the individual at work. Other studies have also shown workers with work-related asthma have impaired physical and mental health, (Mazurek *et al*., 2011; Knoeller *et al*., 2012) have increased need for medical resources, (Lemiere *et al*., 2007) poorer asthma control (Mazurek *et al*., 2011) and worse quality of life (Lowery *et al*., 2007). This small study does not reasonably allow for further inference here. The decrement seen was in the order of 0.05 units of the EQ-5D-related utility index, the QALY equivalent of which could be factored into future economic analyses of costs associated with work-related asthma (Whitehead and Ali, 2010).

There are few previous studies that have assessed the wider impact of work-related asthma symptoms, although the available evidence suggests that WAA is associated with a significant socio-economic impact. For example, unemployment rates have been found to be equal and high (between 31 and 39%) in workers with OA, work aggravated asthma and those with asthma not associated with work (Cannon *et al*., 1995). However, Larbanois *et al* (2002) reported a more frequent reduction in income in those with WAA (65%) and occupational asthma (62%) compared with those workers with asthma unrelated to work (38%). In addition, job change or work loss due to asthma was seen in very high levels for both WAA (54%) compared with those workers with occupational asthma (52%).

### Implications for future research, policy and practice

There are currently no published intervention studies for work aggravated asthma. Future research and clinical work could identify and better assist the understanding of the most significant aggravants to asthma at work. Further work to look at ERI and health inequalities in the workplace may also help us to understand the link between stress and WAA. This would undoubtedly help define appropriate interventions. These interventions would seem appropriate to develop with primary care asthma experts, but also with input from those with knowledge of health at work and the national regulator HSE, and perhaps most importantly in conjunction with workers, their representative, and employers.

## Conclusions

Asthma symptoms that worsen at work (WAA) are commonly reported by workers in GB. The estimate of 33% of those with asthma complaining of work-related symptoms is in line with international prevalence.
